# Characterization of the First Case of Classical Scrapie in a Sheep in Tunisia

**DOI:** 10.1155/2023/2253316

**Published:** 2023-10-25

**Authors:** Abdelkader Amara, Kéfia Elmehatli, Michele Angelo Di Bari, Laura Pirisinu, Rihab Andolsi, Souhir Gachout, Boubaker Ben Smida, Meriem Handous, Heni Haj Ammar, Roukaya Khorchani, Malek Zrelli, Barbara Iulini, Caterina Lucia Florio, Maria Caramelli, Cristina Casalone, Laura De Antoniis, Geraldina Riccardi, Elena Esposito, Matteo Giovannelli, Claudia D'Agostino, Barbara Chiappini, Romolo Nonno, Umberto Agrimi, Gabriele Vaccari

**Affiliations:** ^1^National School of Veterinary Medicine of Sidi Thabet, Manouba University, Tunis, Tunisia; ^2^Animal Production District of Tataouine, Tataouine, Tunisia; ^3^Department of Food Safety, Nutrition and Veterinary Public Health, Italian National Institute of Health, Rome, Italy; ^4^Pasteur Institute of Tunisia, Tunis, Tunisia; ^5^General Directorate of Veterinary Services of Tunisia, Tunis, Tunisia; ^6^Istituto Zooprofilattico Sperimentale del Piemonte Liguria e Valle d'Aosta, Turin, Italy; ^7^Istituto Zooprofilattico Sperimentale dell'Abruzzo e Molise “G. Caporale”, Teramo, Italy

## Abstract

Classical scrapie is a contagious prion disease of sheep and goats. It is endemic in many countries in Europe, North America, and Asia. In Africa, imported scrapie cases have been described in South Africa and Kenia in the past. More recently, several cases have been reported from different regions of Libya, based on clinical signs and histological lesions. Here, we report the results of thorough investigations carried out on a suspect case of scrapie in a 6-year-old Barbarine sheep, born, and bred in Tunisia, showing behavioral changes, weight loss, itching, skin lesions, wool loss, and motor incoordination. Histopathology and immunohistochemistry revealed spongiform change in several brain areas with associated pathological prion protein deposition. Western blotting confirmed the diagnosis and showed a classical scrapie-like molecular pattern of PrP^res^, different from atypical scrapie and bovine spongiform encephalopathy (BSE) in small ruminants. Sequence analysis of the prion protein gene showed that the animal carried the ARQ/ARQ genotype, one of the most susceptible to classical scrapie. The inoculation of sheep brain homogenate in a susceptible rodent model proved the experimental transmissibility of the disease. These results demonstrate the circulation of classical scrapie in Tunisia and confirm its presence in North Africa, indicating the need to improve epidemiological surveillance and diagnostic capacity for prion diseases in the region.

## 1. Introduction

Scrapie of sheep and goats is a neurodegenerative and lethal disease belonging to a group of human and animal diseases known as transmissible spongiform encephalopathies (TSE) or prion diseases which also includes bovine spongiform encephalopathy (BSE) in cattle and Creutzfeldt-Jakob disease in humans [1].

Key event in the pathogenesis of prion diseases is the conformational change of the cellular prion protein (PrP^C^) into a pathological isoform (PrP^Sc^) that accumulates in the brain of affected subjects [1]. Scrapie has been first described in the 18th century in the United Kingdom [2]. Since then, it has been reported worldwide in many countries except for Australia and New Zealand where the disease was eradicated soon after its first appearance caused by the importation of infected sheep in the 1950s [3].

Scrapie exists in two forms, the classical form is a contagious disease under natural conditions, while atypical scrapie/Nor98, described for the first time in Norway in 1998, is believed to be a sporadic disease and there is currently no evidence for it being infectious [4].

Classical scrapie is endemic in many countries in Europe, North America, and Asia and has been found in almost all countries where extensive surveillance has been implemented [5]. It has long incubation periods and usually affects animals between 2 and 5 years. The disease lasts for 1–6 months between appearance of clinical signs and death [6].

Initially, affected animals show behavioral changes, which progress to more obvious neurological signs, such as incoordination of movement, ataxia, collapse episodes, and cachexia. Behavioral changes may vary between affected animals, but the most common are restlessness, hyperesthesia, hyperexcitability to external stimuli, separation from the flock, and itching which can cause areas of alopecia by continuous scratching [5].

The susceptibility of sheep to classical scrapie is strongly influenced by polymorphisms of the PrP gene (*PRNP*) at codons 136, 154, and 171 [7]. The ARQ (Alanine at codon 136, Arginine at 154, and Glutamine at 171) is the archetype PrP allele, and ARQ/ARQ homozygotes animals are among the most susceptible to the disease. Strikingly, homozygous and heterozygous sheep for the ARR (Alanine at codon 136, Arginine at 154, and Arginine at 171) allele (genotypes ARR/ARR and ARR/with other alleles) are resistant to scrapie [8]. This prompted European countries and USA to achieve classical scrapie control and eradication by breeding for genetic resistance targeting the prion protein gene [9].

Epidemiological investigations suggest that the transmission of classical scrapie occurs mainly horizontally, either by direct contact between affected and healthy animals or indirectly through contamination of the environment [10]. Maternal transmission also occurs, and the placenta is thought to be one of the most important sources of infection [11]. Infectivity has been detected in secretions (milk and saliva) [12, 13], feces [14], and semen [15] of scrapie-affected animals. Moreover, the detection of infectious prions in urine of infected rodent models [16] coupled with the demonstration of PrP^Sc^ in the kidney of scrapie-affected sheep suggests the possible excretion of infectivity with urine [17].

Infection is acquired via the oral route and prions localize first in the palatine tonsils and the gut-associated lymphoid tissue [18, 19]. Subsequently, before targeting the central nervous system mainly through the peripheral nervous system, the scrapie agent replicates in macrophages and follicular dendritic cells of the lymphoreticular system and significant depositions of PrP^Sc^ can be detected by immunohistochemistry in the spleen and lymph nodes throughout the body [20].

Confirmatory diagnosis of a scrapie suspect can be achieved only postmortem since it requires laboratory tests on central nervous system samples to be carried out. The detection of PrP^Sc^ in the brain of affected animals by Western blot or enzyme-linked immunosorbent assay (ELISA) represents the most used approach for scrapie confirmation [5]. PrP^Sc^ can be also detected by immunohistochemistry, provided that suitable pretreatments are applied to tissue sections [21].

In Africa, scrapie has been described in 1966 in South Africa [22] and in 1973 in Kenya [23], always in Hampshire Down breed sheep that had been imported from outside the continent. Diagnostic investigations for scrapie have been carried out in Nigeria, but none of the clinical suspects were confirmed as to be affected by the disease [24]. In Libya, scrapie was reported in 2014 to the WOAH [25]. In 2022, nine out fourteen sheep older than 2 years, with neurological signs and originating from several regions of Libya were diagnosed with scrapie based on the spongiform change detected by brain histopathology [26].

Herein, we describe the results of investigations carried out to fully characterize the first case of scrapie reported in Tunisia, in a Barbarine sheep.

## 2. Materials and Methods

### 2.1. Scrapie Suspect and Sampling

A suspect of scrapie was raised in a 6-year-old ewe of the autochthonous Tunisian Barbarin breed, from the Governorate de Tataouine, délégation de Remada, in the south of Tunisia in September 2019. The sheep (here identified as 19/185) was born and grown in Tunisia and fed on pasture, hay, and barley. It was presented to the veterinary services, showing behavioral changes, pruritus, repetitive licking, teeth grinding, deterioration of general conditions, motor incoordination, and paraplegia. The clinical signs lasted 52 days before death. At necropsy, samples of the cervical marrow, hippocampus, and cerebellum were collected and sent to the Pasteur Institute in Tunis, for rabies diagnosis, which turned out negative. Portions of the obex and cerebral cortex, and half of the cerebellum and lymph nodes were partly fixed in formalin and partly frozen.

### 2.2. Histopathology and Immunohistochemistry

Formalin-fixed brain samples as well as retropharyngeal, prescapular, and popliteal lymph nodes from the sheep suspected to be affected by scrapie were embedded in paraffin wax, sectioned at 5 *μ*m and stained with hematoxylin and eosin or subjected to immunohistochemistry. The brains from experimentally inoculated voles were processed as previously described [27].

Sections for immunohistochemistry were pretreated with 98% formic acid for 5 min, followed by autoclaving in citrate buffer for 5 min at 121°C. Sections were then treated with 6% normal goat serum (Vector Laboratories) in PBS for 1 hr. Immunohistochemical detection of PrP^Sc^ of sheep samples was performed with L42 mAb (R-Biopharm) at 0.01 *µ*g/ml, while vole brain sections were analyzed using SAF84 mAb (Bertin-Pharma) at 0.3 *µ*g/ml. Astrogliosis in vole brains was detected using PA5 anti-Glial Fibrillary Acidic Protein (GFAP) antibody (Invitrogen) at 5 *µ*l/ml. Primary antibodies were incubated overnight at 4°C. Sections were treated with secondary biotinylated antimouse antibody (Vector), ABC Complex (Vector) for 45 min. Finally, the sections were stained with diaminobenzidine (Dako-Cytomation) and counterstained with Mayer's hematoxylin.

### 2.3. Western Blotting

Western blot analysis of PrP^res^ (the resistant core of PrP^Sc^ after proteinase treatment) was performed from sheep brain homogenates according to the ISS discriminatory Western blotting method approved for BSE and scrapie surveillance in Europe [28]. Briefly, brain homogenates 20% (wt/vol) were prepared in 100 mM Tris-HCl with complete protease inhibitor cocktail (Roche) at pH 7.4. After adding an equal volume of 100 mM Tris-HCl containing 4% sarkosyl, the homogenates were incubated for 30 min at 25°C with gentle shaking. Proteinase K (Sigma–Aldrich) was added to a final concentration of 200 *µ*g/ml. The reaction was stopped with 3 mM PMSF (Sigma–Aldrich). Aliquots of samples were added with an equal volume of isopropanol/butanol (1 : 1 vol/vol) and centrifuged at 20,000 × *g* for 10 min. The pellets were resuspended in denaturing sample buffer (NuPAGE LDS Sample Buffer; Life Technologies) and heated for 10 min at 90°C. We loaded each sample onto two 12% bis-Tris polyacrylamide gels (Invitrogen) for electrophoresis with subsequent western blotting on polyvinylidene fluoride membranes using the Trans-Blot Turbo Transfer System (Bio-Rad) according to the manufacturer's instructions. The blots were processed with anti-PrP mAbs P4 (epitope at aa 93–99, sheep PrP numbering) and SAF84 (aa 167–173) using the SNAP i.d. 2.0 system (Millipore) according to the manufacturer's instructions. After incubation with horseradish peroxidase-conjugated anti-mouse immunoglobulin (Pierce Biotechnology) at 1 : 20,000, the PrP bands were detected using enhanced chemiluminescent substrate (SuperSignal Femto; Pierce Biotechnology) and ChemiDoc imaging system (Bio-Rad). The molecular weight of the nonglycosylated PrP^res^ band and the signals obtained by the two mAbs were quantified using Image Lab Software (Bio-Rad).

The principle of discrimination of scrapie from BSE is based on the differential N-terminal cleavage by PK, revealed using N-terminal mAb (P4) with an epitope that is partially lost after PK digestion of BSE samples [28]. Two cut-off values for BSE are applied: in BSE samples the P4/SAF84 ratios should be >2 and the molecular mass of the nonglycosylated PrP^res^ band based on SAF84 <0.5 kDa, compared to that of internal classical scrapie control. Glycoform profiles, that is, the relative proportion of diglycosylated, monoglycosylated, and unglycosylated PrP^res^ fragments were quantified in SAF84 blot by Image Lab software (Bio-Rad).

PrP^res^ from vole brain homogenates was analyzed as previously described [29].

### 2.4. Sequencing Analysis

Sequencing analysis was carried out at the Istituto Superiore di Sanità.

DNA was extracted from 100 mg of frozen sheep brain tissue with DNeasy Blood and Tissue Kit (QIAGEN, Hilden, Germany) following the manufacturer's instructions.

*PRNP* coding sequence (CDS) was amplified using 5 *µ*l of extracted DNA, 1X AmpliTaq Gold® 360 PCR Buffer, 2.5 mM MgCl_2_, 1X 360 GC Enhancer, 200 *µ*M dNTPs, 0.25 *µ*M of F1 (5ʹ-CATTTATGACCTAGAATGTTTATAGCTGATGCCA-3ʹ) and R1 (5ʹ-TTGAATGAATATTATGTGGCCTCCTTCCAGAC-3ʹ) primers and 0.5 *µ*l di AmpliTaq Gold® 360 5 U/*µ*l, (Applied Biosystems), following standard amplification protocol (5ʹ at 96°C; 30ʺ at 96°C; 15ʺ at 57°C; 90ʺ at 72°C for 35 cycles; and 4ʹ 72°C).

Amplicons were purified using Illustra ExoProStar 1-Step clean-up kit (GE Health care Life Sciences AB). Sequencing reactions were carried out with 0.32 *µ*M of primers T3 (5ʹ-TTT ACG TGG GCA TTT GAT GC-3ʹ) and T4 (5ʹ-GGC TGC AGG TAG ACA CTC C-3ʹ) using Big Dye Terminator Cycle sequencing Kit v.1.1 (Life Technologies), subsequently purified using BigDye XTerminator Purification kit and detected with ABI PRISM 3130 apparatus (Applied Biosystems). Sequences were analyzed using the software SeqScape v.2.5 (Applied Biosystems).

### 2.5. Experimental Transmission to Bank Voles

Fourteen bank voles (*Myodes glareolus*) carrying methionine at codon 109 of PrP, a rodent model very susceptible to different scrapie strains [27, 30–35], were intracerebrally inoculated with 20 *µ*l of 10% sheep brain homogenate in phosphate-buffered saline. The inoculation was carried out under general gaseous anesthesia (Isofluorane 5%). The animals were examined twice a week until the appearance of neurological signs, and daily thereafter. Diseased animals were culled in a 100% saturated carbon dioxide room at the terminal stage of the disease, but before the severity of neurological impairment compromised their welfare, in particular, their ability to drink and feed adequately. Immediately at postmortem, each brain was removed and divided into two parts by a sagittal paramedian cut. The smaller portion (left part) was immediately stored at −80°C for biochemical investigation, while the larger one (right part) was fixed in 10% neutral buffered formalin.

### 2.6. Ethics Statement

Experiments involving animals adhere to the Legislative Decree 26/2014, which transposes in the Italian legislation the European Directive 2010/63/EU on Laboratory Animal Protection. The experimental protocols were approved and supervised by the Animal Welfare Service of the Istituto Superiore di Sanità and were authorized by the Italian Ministry of Health (decree number 1115/2019-PR).

## 3. Results and Discussion

**Figure 1:**
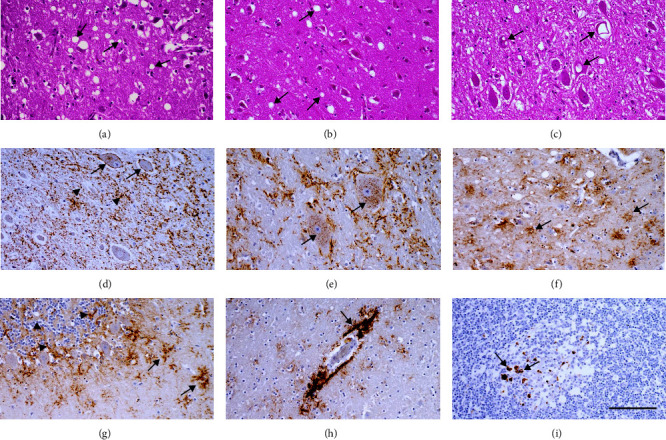
Hematoxylin and eosin staining (a–c) and PrP^Sc^ immunohistochemistry (d–h) of brain or retropharyngeal lymph node (i) of the sheep 19/185. Spongiform change of the neuropil (arrows) in the mediodorsal thalamic nucleus (a); spongiosis (arrows) of the neuropil in the deep layers of occipital cortex (b) and intraneuronal vacuoles (arrows) in the dorsal nucleus of the vagus nerve (c). Main PrP^Sc^ deposition patterns observed in the brain (d–h). Hypoglossal nucleus: perineuronal and intraneuronal (arrows) and synaptic/punctate (arrowheads) in the neuropil (d); intraneuronal accumulation in the reticular formation of medulla oblongata (arrows) (e); stellate deposition in the white matter of the occipital cortex (f); stellate (arrows) and small PrP^Sc^ deposits (arrowheads) in the molecular and granular layer of the cerebellum, respectively (g); perivascular in the white matter of the occipital cortex (h). Retropharyngeal lymph node (i): granular and punctuate PrP^Sc^ depositions in the germinal centre of a secondary lymphoid nodule (i) (scale bar for all figures of the panel, 50 *µ*m).

Histopathological examination of the brain of sheep 19/185 revealed spongiform change, gliosis and neuronal loss in different gray matter areas. In particular, moderate neuropil vacuolation affected basal ganglia, thalamus (Figure 1(a)), midbrain, and pons, while hippocampus and cerebellum showed rare vacuoles. Moderate spongiform change was also visible in the neocortex, affecting both deep and superficial layers (Figure 1(b)). We observed intraneuronal vacuolation in different nuclei of the obex, such as dorsal nucleus of the vagus nerve (Figure 1(c)), hypoglossal, and olivary nucleus.

Pathological prion protein deposition was found widespread in all brain areas, involving both gray and white matter. Overall, PrP^Sc^ deposition roughly overlapped the distribution of spongiform change. The main patterns of PrP^Sc^ deposition, included perineuronal and intraneuronal (Figures 1(d) and 1(e)), associated with astrocytes as stellate pattern (Figures 1(f) and 1(g)), synaptic and punctuate in the neuropil (Figures 1(d) and 1(f)), and perivascular (Figure 1(h)).

In the retropharyngeal, prescapular, and popliteal lymph nodes, PrP^Sc^ immunolabeling was detected by immunohistochemistry as a granular and punctuate deposition mainly located in the germinal centers of secondary nodules (Figure 1(i) and Figure S1). Although no specific analyses were performed to identify the cells involved, PrP^Sc^ staining appeared associated to cells whose morphology was consistent with macrophages.

**Figure 2:**
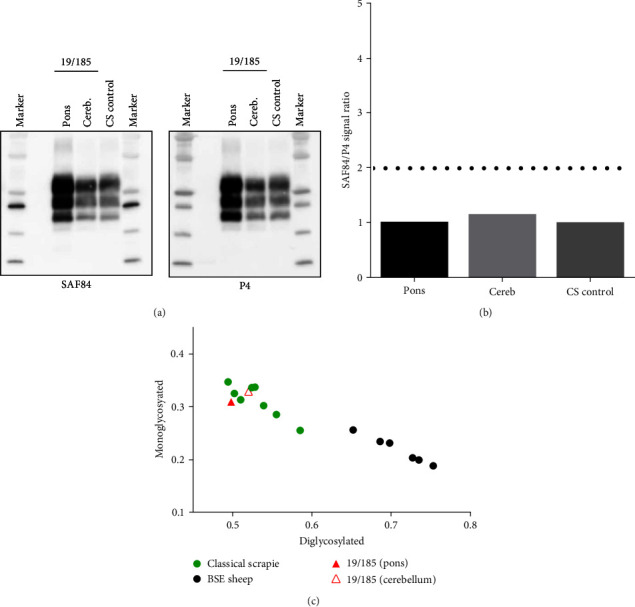
PrP^res^ analyses of pons and cerebellum samples from scrapie-affected sheep from Tunisia show characteristics (i.e., molecular weight of the nonglycosylated band (a); SAF84/P4 ratio relative to the scrapie control (b); proportions of diglycosylated and monoglycosylated PrP^res^ bands (c)) comparable to classical scrapie: (c). (a) Western blot analysis of proteinase K (PK)—treated PrP^Sc^ in brain homogenates from a suspect case of sheep scrapie, Tunisia. Two brain areas (pons and cerebellum) from case 19/185 were analyzed along with an isolate of classical scrapie (CS) from an Italian sheep (indicated as CS control). According to ISS-WB protocol, the membranes were probed with SAF84 and P4 monoclonal antibodies (as indicated on the bottom of the blots). The molecular weight markers (Biorad) were loaded in each gel (Marker) indicating 10, 15, 20, 25, 37, and 50 kDa. (b) Bar graph showing the antibody ratio (SAF84/P4 ratio) of the chemiluminescence signal for the samples of pons and cerebellum from case 19/185 shown in (a), relative to the SAF84/P4 ratio of the control scrapie, according to ISS-WB. The horizontal dashed line refers to the cut-off value of the antibody ratio, according to the ISS-WB protocol (antibody ratio 2). (c) Scattergraph of proportions of diglycosylated and monoglycosylated PrP^res^ bands from sheep case 19/185 in comparison to those previously reported for small ruminants with classical scrapie and BSE [36]. In green are indicated classical scrapie isolates, in black the samples from sheep experimentally infected with BSE, and in red the two samples from brain of sheep 19/185.

Samples from pons and cerebellum were analyzed by Western blot and compared with a brain sample from a sheep belonging to an Italian outbreak of classical scrapie. The analysis showed a PrP^res^ characterized by the typical three-bands pattern (Figure 2(a)) and a similar amount of PrP^res^ with both antibodies. Indeed, the antibody ratio (SAF84/P4) of PrP^res^ from the pons and the cerebellum relative to the SAF84/P4 ratio of the control scrapie was 1.0 and 1.2, respectively (Figure 2(b)). The molecular weight of unglycosylated band of PrP^res^ was ≈17.6 kDa, in the range of molecular weight of classical scrapie control (17.5–17.9 kDa).

The glycoform ratio (relative proportions of diglycosylated, monoglycosylated, and unglycosylated bands) can be used as an additional discriminatory parameter [36, 37]. The samples from both brain areas of case 19/185 showed a diglycosilated-to-monoglycosilated glycoform ratio (0.50 : 0.30 and 0.52 : 0.32) similar to classical scrapie cases (range from 0.50 : 0.30 to 0.58 : 0.25) and less glycosylated than expected for BSE in small ruminants (range from 0.70 : 0.23 to 0.75 : 0.19) (Figure 2(c)). The molecular pattern of this case was also different from atypical/Nor98 scrapie that shows a characteristic banding with a prevalent band of ∼12 kDa [38, 39].

Besides the molecular features of PrP^res^, the overall neuropathological picture and PrP^Sc^ deposition in lymphoid tissue converge to classify this as a case of classical scrapie. Indeed, in atypical scrapie/Nor98, vacuolization and PrP^Sc^ deposition mainly occur in the white matter and in brain areas different from classical scrapie, such as in the cerebellar and cerebral cortex and are modest in the brainstem which is a target area of classical scrapie [40]. Moreover, the deposition of PrP^Sc^ in the brain of atypical scrapie/Nor98 scrapie cases is always extracellular and shows a rather uniform punctuate pattern. Conversely, PrP^Sc^ deposition in classical scrapie exhibits a variety of patterns, both intracellular and extracellular. Finally, PrP^Sc^ is undetectable in lymphoid tissue of atypical/Nor98 scrapie cases, while it represents a common finding in classical scrapie [41].

**Figure 3:**
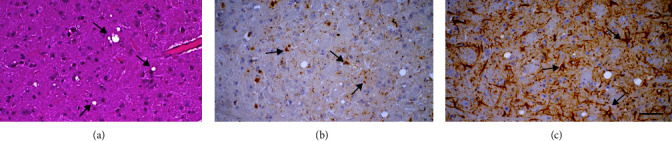
Serial sections of medulla oblongata from a vole experimentally infected with Tunisian scrapie, analyzed with hematoxylin eosin (a), and immunohistochemistry for PrP^Sc^ (b), or GFAP (c). Vacuolization (arrows) (a), and fine/coarse granular PrP^Sc^ deposits (arrows) (b), dense astrocytic gliosis (arrows) (c) (scale bar, 20 *µ*m).

A key characteristic of prion diseases is their experimental transmissibility. Upon inoculation, all voles developed overt clinical signs of disease and showed survival time of 183 ± 23 days postinoculation. Clinical signs in voles began from mild behavioral alterations and the disappearance of the typical behavior of hiding under the sawdust lining the cage at disease onset to dorsal kyphosis, severe ataxia, and head bobbing in full-blown disease. Neuropathological analysis of voles showed intense spongiform change, PrP^Sc^ depositions, and astrogliosis in several areas, particularly in the superior colliculus, thalamus, hypothalamus, and neocortex (Figure 3). The successful transmission to voles was also confirmed by the PrP^res^ detection in brain homogenates by Western blot. Bioassay results demonstrate the transmissibility of the prion agent responsible for this scrapie case. Moreover, given the substantial resistance of bank voles to atypical scrapie/Nor98 [42], the easy transmission of this case further confirms the diagnosis of classical scrapie.

Our sequencing analysis revealed that the affected sheep carried the wildtype ARQ/ARQ genotype, which is susceptible to classical scrapie and frequent in the Barbarine breed (43.1%) [43]. Nevertheless, the ARR allele that is associated with resistance to classical scrapie is the second most frequent following the ARQ [43], thus representing a good potential basis for genetic resistance in this breed.

While there is no direct evidence that scrapie can be transmitted to humans [44], the potential of scrapie to infect humans has been reported in mouse models [45]. However, after the emergence of BSE and variant CJD, a prion disease of humans linked to the consumption of BSE prion-contaminated food [46], prion diseases in animals have become a major concern. Moreover, the hypothesis that BSE could circulate, unrecognized, in the European sheep population which had been exposed to BSE-infected feed [47], has led Europe and the United States to launch severe scrapie control programs.

Molecular investigations and bioassays in rodent models allowed to establish that at least four different strains of classical scrapie do exist [48–50]. As regard the prion involved in the Tunisian scrapie case, its molecular signature was typical of classical scrapie and different from both BSE and atypical scrapie. Bioassays in rodent models are ongoing for the full characterization of the prion strain and for its comparison with scrapie cases from Europe.

Scrapie can cause severe economic damage to the sheep industry. Within the United States alone, it brings economic losses due to decreased production, export loss, and increased cost for carcass disposal of up to US $20 million annually [51].

The management of prion diseases encounters serious limitations. No vaccine or therapy do exist. To date, the only successful strategy against the disease is the genetic selection of sheep for resistance traits as demonstrated by the dramatic decrease of cases observed in some European countries where the genetic selection has been pursued more efficiently [9].

Sheep and goats are important economic resources for Tunisia and sheep red meat production represents ∼45% [52] of the total red meat production in Tunisia. The scrapie case described here came from the southern and driest region of the country where sheep and goats often represent a vital resource for people. In that area, extensive grazing and transhumance are practiced, which facilitate long-distance contacts between animals of different flocks and produce favorable conditions for scrapie to spread.

The results of this study demonstrate the autochthonous circulation of classical scrapie in sheep in Tunisia. This finding, along with the recent report of scrapie cases from Libya [26] and the description of a novel prion disease in dromedary in Algeria [53] suggest the need to strengthen epidemiological surveillance and improve the diagnostic capacity for animal prion diseases in North African countries.

## Data Availability

Data available on request.

## Supplementary Materials

Supplementary Materials Figure S1: granular and punctuate PrP^Sc^ depositions (arrows) in the germinal centre of secondary lymphoid nodules of prescapular (a) and popliteal (b) lymph nodes.
