# Supplementary figures and images for: Characterization of the First Case of Classical Scrapie in a Sheep in Tunisia
Source: Transbound Emerg Dis. 2023 Oct 25;2023:2253316. doi: 10.1155/2023/2253316 (PMC12017096; doi:10.1155/2023/2253316)

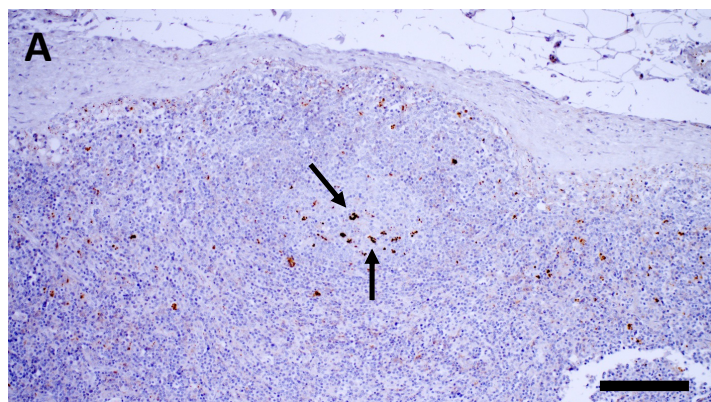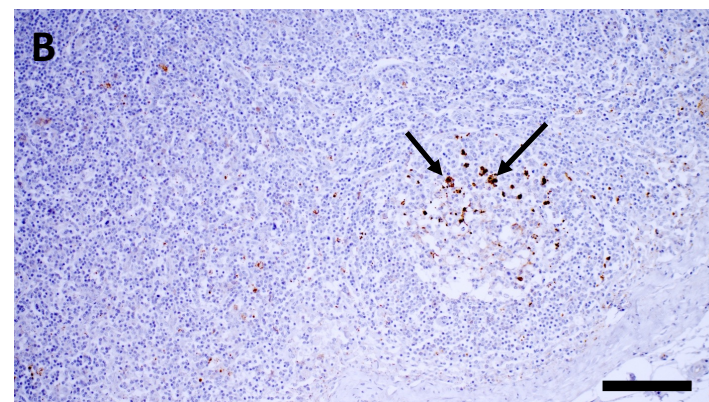

Supplement: Supplementary Materials — Figure S1: granular and punctuate PrPSc depositions (arrows) in the germinal centre of secondary lymphoid nodules of prescapular (a) and popliteal (b) lymph nodes. [file 2253316.f1.pdf]
